# Post-operative C-reactive protein as a strong independent predictor of long-term colorectal cancer outcomes: consistent findings from two large patient cohorts

**DOI:** 10.1016/j.esmoop.2024.102982

**Published:** 2024-04-12

**Authors:** T. Gwenzi, P. Schrotz-King, S.C. Anker, B. Schöttker, M. Hoffmeister, H. Brenner

**Affiliations:** 1 Division of Preventive Oncology, German Cancer Research Center (DKFZ) and National Center for Tumor Diseases (NCT), Heidelberg; 2 Medical Faculty Heidelberg, Heidelberg University, Heidelberg; 3 Department of Internal Medicine, University Hospital Heidelberg, Heidelberg; 4 Division of Clinical Epidemiology and Aging Research, German Cancer Research Center (DKFZ), Heidelberg; 5 Network Aging Research, Heidelberg University, Heidelberg; 6 German Cancer Consortium (DKTK), German Cancer Research Center (DKFZ), Heidelberg, Germany

**Keywords:** colorectal, C-reactive protein, survival, systemic inflammation, prognosis

## Abstract

**Background:**

Post-surgery blood-based biomarkers may be useful for guiding treatment and surveillance decisions among colorectal cancer (CRC) patients. However, most candidate biomarkers provide little if any predictive value beyond stage at diagnosis. We aimed to investigate the independent prognostic value of post-operative serum C-reactive protein (CRP), a highly sensitive biomarker of inflammation, for long-term CRC outcomes in two large patient cohorts.

**Materials and methods:**

CRP levels were measured from serum samples of CRC patients collected ≥1 month post-surgery in the German DACHS (*n* = 1416) and the UK Biobank (*n* = 1149) cohorts. Associations of post-operative CRP with overall survival (OS) and CRC-specific survival (CSS) were assessed using Cox regression and presented as hazard ratios (HRs) with 95% confidence intervals (CIs), adjusted for key sociodemographic and clinical covariates.

**Results:**

In both cohorts, consistent strong dose–response relationships between post-operative CRP and both OS and CSS were observed. Adjusted HRs (95% CI) for CRP >10 versus <3 mg/l were 1.93 (1.58-2.35) and 2.70 (2.03-3.59) in the DACHS cohort, and 2.70 (1.96-3.71) and 2.61 (1.83-3.72) in the UK Biobank cohort, respectively. Associations between post-operative CRP and OS were particularly strong among younger patients (<65 years at diagnosis; *P* value for interaction by age <0.01).

**Conclusions:**

Serum CRP determined a month or more after surgery may be useful as a strong independent prognostic biomarker for guiding therapeutic decisions and for surveillance of the course of disease of CRC patients, particularly those <65 years of age at diagnosis.

## Highlights

• Patients were treated with surgery for CRC.
• Post-operative CRP (poCRP) was measured from blood collected ≥1 month after surgery.
• Associations of poCRP with long-term survival were assessed using Cox regression.
• Elevated poCRP was independently associated with poorer survival.
• poCRP assessed ≥1 month after surgery may be valuable for CRC surveillance.

## Introduction

Colorectal cancer (CRC) is the second leading cause of cancer death and the third most diagnosed cancer globally.^1^ Prognosis of patients with CRC strongly depends on stage at diagnosis, with 5-year relative survival ranging from >90% for patients with localised disease to <15% for patients with distant metastases,^2^ which underlines the large potential of reducing the burden of the disease by effective screening programmes.^3^ Nevertheless, there is considerable heterogeneity in survival outcomes also for CRC patients within the same stage.^4^ Besides major variation of prognosis according to stage, site and molecular features of the tumour,^5^ additional patient factors may be informative to guide treatment and surveillance decisions. Although many candidate biomarkers have been proposed and evaluated, most of them provide very limited prognostic value beyond stage at diagnosis.

Inflammation is a notable hallmark of cancer with strong links to both tumorigenesis and tumour progression.^6^, ^7^, ^8^ Circulating C-reactive protein (CRP) is a well-established and highly sensitive biomarker of systemic inflammation that has been shown to be associated with survival of patients with various diseases including cancer.^9^ Elevated post-surgery serum levels of CRP among CRC patients have been linked to shorter survival, independent of tumour stage at diagnosis.^10^^,^^11^ However, pertinent evidence is mostly based on small studies with partly conflicting results.^12^^,^^13^ Since systemic inflammation is highly variable and strongly triggered by the acute care interventions or its potential complications in the immediate period following surgery,^14^ this study seeks to thoroughly investigate the association of serum CRP concentrations assessed a month or more after surgery with survival outcomes of CRC patients in two independent cohorts, the German DACHS study and the UK Biobank.

## Materials and methods

### Study populations and study design

**Figure 1:**
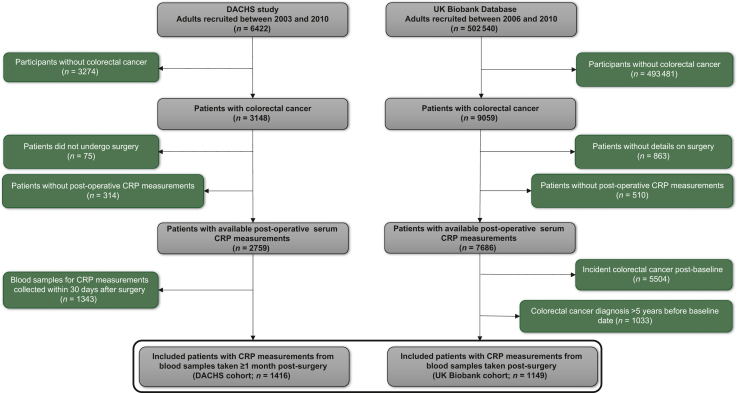
Study participant selection flow diagram for the DACHS and UK Biobank cohorts. CRP, C-reactive protein.

Figure 1 shows the details of patient selection criteria. We retrospectively analysed data from two population-based cohort studies prospectively recruiting participants in Germany (DACHS study) and in the UK (UK Biobank), both following the guidelines of the Declaration of Helsinki. The DACHS study is a population-based case-control study with long-term follow-up of patients (≥30 years of age, no upper age limit) who were recruited after a first diagnosis of CRC from 22 clinics in the Rhine-Neckar region in south-west Germany in 2003-2021. Details of the study design, recruitment, data collection and follow-up procedures have been reported elsewhere.^15^, ^16^, ^17^, ^18^, ^19^

Briefly, standardised questionnaires were used to collect sociodemographic, lifestyle history and medical information from CRC patients by personal interviews conducted during hospital stay for surgery or within weeks to months after discharge. Medical data on tumour stage and site, and therapy were obtained from hospital charts. Blood samples were collected after personal interviews and serum aliquots were stored at −80°C until analysis. Mortality follow-up was conducted through record linkage with population registries, and cause of death was obtained from health authorities through death certificates using the 10th revision of the International Statistical Classification of Diseases (ICD-10) to identify mortality due to CRC (C18-C20). The study was approved by the ethics committees of the University of Heidelberg (#310/2001, 06 December 2001) and the state medical boards of Baden-Württemberg and Rhineland-Palatinate. All participants provided written informed consent. For the current analysis, we included a total of 1416 patients with CRC recruited in 2003-2010 for whom blood samples taken at least 1 month post-surgery and long-term follow-up data with respect to survival were available.

The UK Biobank is a prospective cohort study that recruited over 500 000 men and women aged between 40 and 69 years in 2006-2010. Biomedical information was collected from the 22 assessment centres across England, Scotland and Wales through questionnaires, verbal interviews, physical and medical assessments. Biological specimens such as blood, urine, stool and hair were collected at the initial assessment visit (baseline date).^20^ Data on health outcomes were gathered through linkages to health care records, including the UK National Health Service (NHS) data, primary care data, cancer screening data and disease-specific registers.^21^ Information on the dates and causes of death was obtained from the NHS for the period of time from enrolment to 12 November 2021. The ICD-10 was used to identify causes of death. The UK Biobank was approved by the North West Multi-Center Research Ethics Committee (MREC) as a research tissue bank (approval renewed in 2021: 21/NW/0157). The UK Biobank study was approved by the North West Haydock Research Ethics Committee (#16/NW/0274, 13 May 2016). For the current analysis, we included 1149 patients with a CRC diagnosis ≤5 years before the baseline date, for whom CRP measurements and follow-up details of survival outcomes of interest were available. From the available UK Biobank data, all participants had their blood samples collected at least 9 months after surgery; therefore, complete exclusion of patients with CRC surgery <1 month before recruitment or blood sampling was not necessary.

### Post-operative CRP measurements

For the DACHS cohort, the ADVIA XPT Siemens Healthineers instrument (Siemens Healthineers AG, Erlangen, Germany) was used to measure CRP in 300 μl post-operative serum samples by immunoturbidimetric method. Measurements for serum CRP were conducted over a time span of 3 months in 2022 at the Central Laboratory of the Heidelberg University Clinics. In the UK Biobank cohort, CRP was measured at baseline within 24 h of the blood draw during recruitment. CRP was measured using the immunoturbidimetric method with the Beckman Coulter AU5800 instrument (Beckman Coulter, Inc., Brea, CA).^21^ In both cohorts, CRP assays were conducted blinded to the survival endpoints. CRP levels <3 mg/l are generally known to be reflective of good health and disease control^22^; therefore, CRP was converted into a variable with four categories comparing patients with CRP levels of 3-5 mg/l, 5-10 mg/l and ≥10 mg/l to the reference group of patients with CRP levels <3 mg/l.

### Outcomes

Endpoints for overall survival (OS) and CRC-specific survival (CSS) were defined as death from any cause or death from CRC, respectively. In addition, for the DACHS cohort, we also estimated the relapse-free survival (RFS). Follow-up times for survival outcome endpoints were calculated in days beginning from the date of blood sampling (≥1 month and up to 52.6 months after surgery in the DACHS cohort and up to 60 months after surgery in the UK Biobank cohort) to the date of having the event. Patients who did not reach a specific endpoint were censored at the time when they were last known to have been alive.

### Covariates

For our analyses, we considered the following factors as covariates for both cohorts: sex, age at diagnosis, body mass index (BMI), alcohol consumption, history of smoking, physical exercise, history of cardiovascular disease, history of diabetes, history of hypertension, vitamin D status and season of blood draw. Details on TNM (tumour–node–metastasis) stage, chemotherapy use and molecular characteristics [microsatellite stability (MS), *BRAF* and *KRAS* status] were only available in the DACHS cohort at the time of analysis.

### Statistical analyses

Descriptive statistics were used to analyse population characteristics. Continuous variables of patient characteristics are presented as median values with interquartile ranges, while categorical variables are presented as numbers with percentages. For missing values, multiple imputation with five imputed datasets and 30 iterations using the R software ‘mice package’ was used to fill in missing values except for CRP, time to event and survival outcomes.^23^

The distribution of post-operative serum CRP by time after surgery is presented as boxplots. Survival estimates were calculated using Kaplan–Meier methodology and log-rank testing for different categories of CRP. Associations of CRP with survival outcomes were assessed using Cox proportional hazards (PH) models to calculate hazard ratios and 95% confidence intervals, adjusting for all other cohort-specific covariates. The PH assumption was tested by plotting the smoothed Schoenfeld residuals for all covariates against time, and visually assessed for any systematic trends over time. In order to explore the relevance of adjustment for stage, which was not included in the UK Biobank dataset, the analyses of the DACHS data were conducted both with (model 2) and without (model 1) inclusion of stage among the covariates.

We also carried out subgroup analyses by sex, age at diagnosis, BMI, vitamin D status and additionally for the DACHS cohort, cancer stage at diagnosis, chemotherapy use, tumour site (right: cecum to transverse colon; left: splenic flexure to rectum), MS and *BRAF/KRAS* status. In the UK Biobank cohort, blood-cell count variables were available and Cox regressions were additionally adjusted for lymphocyte/monocyte ratio, platelet/lymphocyte ratio and neutrophil/lymphocyte ratio. We assessed potential interactions between CRP and covariates with respect to survival by adding their product term to the Cox PH models, using Wald test statistics for these product terms. We also carried out sensitivity analyses in which follow-up was restricted to 5 years from blood sampling. Statistical analyses were carried out using R statistical software (version 4.2, R Core Team 2023, Vienna, Austria). Two-sided *P* values <0.05 were considered statistically significant for all analyses.

## Results

### Patient characteristics

The main characteristics of patients in the DACHS and UK Biobank cohorts are summarised in Table 1. A total of 1416 and 1149 patients with CRC were included in the DACHS and UK Biobank cohorts, respectively. After a median follow-up of 9.9 years for the DACHS cohort, 690 (48.7%) patients had died, of whom 332 had died from CRC. The median follow-up time in the UK Biobank cohort was 12.2 years during which 317 (27.6%) deaths were reported, 242 of these due to CRC. Both cohorts included more male (61.0% and 59.2%, respectively) than female patients. Median age and median post-operative CRP concentrations were higher in the DACHS cohort (69 years; 2.6 mg/l) than in the UK patient cohort (63 years; 2.2 mg/l). Approximately one half (46.7%) and slightly more than a third (38.1%) of DACHS and UK Biobank patients had post-operative serum CRP concentrations ≥3 mg/l, respectively. Post-operative serum CRP levels after surgery were significantly elevated among patients whose blood samples were collected within the first month following surgery, who were excluded from our analysis on the prognostic value of CRP; thereafter lower and stable median values of CRP were observed for all the successive time periods (Supplementary Figure S1, available at https://doi.org/10.1016/j.esmoop.2024.102982). Moreover, details of cancer stage were only available for the DACHS cohort in which the majority of patients had stage II (32.2%) or stage III (33.4%) CRC at diagnosis.

**Table 1:** Main characteristics of the study population from the DACHS and UK Biobank cohorts

Characteristic	DACHS cohort (*n*_total_ = 1416)	UK Biobank cohort (*n*_total_ = 1149)	*P* value^a^
	*n* (%)	*n* (%)	
Sex			
Female	553 (39.0)	469 (40.8)	0.18
Male	863 (61.0)	680 (59.2)	
Age (years)			
Median (IQR)	69 (61-75)	63 (58-66)	
<60	290 (20.7)	356 (31.0)	**<0.001**
60-64	246 (17.6)	422 (36.8)	
≥65	864 (61.7)	370 (32.2)	
C-reactive protein (mg/l)			
Median (IQR)	2.6 (1.0-7.5)	2.2 (1.1-4.6)	
<3	754 (53.3)	712 (62.0)	**<0.001**
3-5	165 (11.7)	181 (15.8)	
5-10	223 (15.8)	150 (13.1)	
≥10	274 (19.4)	106 (9.2)	
Body mass index (kg/m^2^**)**			
Median (IQR)	26.1 (23.6-29.0)	27.3 (24.0-29.9)	
Normal (18.5-<25)	536 (37.9)	292 (25.4)	**<0.001**
Overweight (25-<30)	608 (42.9)	530 (46.1)	
Obese (≥30)	272 (19.2)	327 (28.5)	
Alcohol consumption			
Yes	1016 (71.8)	762 (66.3)	**0.01**
No	400 (28.2)	387 (33.7)	
History of smoking			
Yes	797 (56.3)	654 (56.9)	0.11
No	619 (43.7)	495 (43.1)	
Physical exercise			
Yes	931 (65.8)	896 (78.0)	<0.001
No	485 (34.2)	253 (22.0)	
Cardiovascular disease			
Yes	334 (24.1)	113 (9.8)	<0.001
No	1053 (75.9)	1136 (90.2)	
History of diabetes			
Yes	258 (18.2)	114 (9.9)	**<0.001**
No	1158 (81.8)	1035 (90.1)	
History of hypertension			
Yes	746 (52.7)	408 (35.5)	**<0.001**
No	670 (47.3)	741 (64.5)	
Serum vitamin D (nmol/l)			
Median (IQR)	29.3 (17.4-45.2)	43.9 (31.3-57.0)	
<30	728 (51.4)	264 (23.0)	**<0.001**
30-49	408 (28.8)	494 (43.0)	
≥50	280 (19.8)	391 (34.0)	
Season of blood draw			
Autumn	334 (24.2)	269 (23.4)	0.72
Spring	396 (28.7)	329 (28.6)	
Summer	348 (25.2)	311 (27.1)	
Winter	301 (21.8)	240 (20.9)	
UICC cancer stage (TNM)			
I	330 (23.3)	NA	NA
II	456 (32.2)	NA	
III	473 (33.4)	NA	
IV	157 (11.1)	NA	
Median follow-up (years)			
Median (IQR)	9.9 (4.8-10.7)	12.2 (11.0-13.2)	
Survival			
Alive	726 (51.3)	832 (72.4)	<0.001
Dead	690 (48.7)	317 (27.6)	
CRC death	332 (23.5)	242 (21.1)	

Missing values were excluded from percentage calculations. Missing data at baseline for age at diagnosis (DACHS = 9, UK Biobank = 2); BMI (UK Biobank = 6); history of cardiovascular disease (DACHS = 29); season of blood draw (DACHS = 137). Season of blood draw: spring: ‘March-May’; summer: ‘June-August’; autumn: ‘September-November’; winter: ‘December-February’.

BMI, body mass index; IQR, interquartile range; NA, not available; TNM, tumour–node–metastasis; UICC**,** Union for International Cancer Control.

a *P* values based on Pearson chi-square test (bold values are statistically significant).

### Post-operative serum CRP levels and survival outcomes

Survival curves for different categories of post-operative serum CRP levels are presented in Supplementary Figure S2, available at https://doi.org/10.1016/j.esmoop.2024.102982. OS and CSS were lower for all categories in which CRP was >3 mg/l compared to CRP <3 mg/l in both cohorts (log-rank *P* value <0.0001), with clear dose–response patterns. Multivariable Cox PH regression results confirmed these strong dose–response patterns in both cohorts (Table 2), which were only slightly attenuated and remained highly statistically significant (*P*_trend_ < 0.001 for both OS and CSS) after additional adjustment for stage at diagnosis in the DACHS cohort. Post-operative CRP was also a strong and independent prognostic factor for RFS in the DACHS cohort (Supplementary Table S1, available at https://doi.org/10.1016/j.esmoop.2024.102982). Besides, additional adjustment for blood-cell count-based inflammatory biomarkers in the UK Biobank showed only slight attenuation for the association of post-operative CRP with both OS and CSS (Supplementary Table S2, available at https://doi.org/10.1016/j.esmoop.2024.102982). Schoenfeld tests showed no violations of PH assumptions for all variables (all *P* values >0.05).

**Table 2:** Multivariable Cox regression results for the association of C-reactive protein with survival outcomes for the DACHS and UK Biobank cohorts

Outcome	DACHS cohort				*P*_trend_	UK Biobank cohort				*P*_trend_
	C-reactive protein serum level					C-reactive protein serum level				
	<3 mg/l	3-5 mg/l	5-10 mg/l	≥10 mg/l		<3 mg/l	3-5 mg/l	5-10 mg/l	≥10 mg/l	
Overall survival										
No. at risk/events	754/315	165/73	223/129	274/171		712/159	181/50	150/51	106/51	
Model 1 HR (95% CI)^a^	**Ref**	1.26 (0.97-1.63)	**1.76 (1.42-2.18)**	**2.01 (1.65-2.45)**	**<0.001**	**Ref**	1.26 (0.92-1.74)	**1.69 (1.23-2.31)**	**2.70 (1.96-3.71)**	**<0.001**
Model 2 HR (95% CI)^b^	**Ref**	1.08 (0.83-1.40)	**1.66 (1.34-2.07)**	**1.93 (1.58-2.35)**	**<0.001**	**—**	**—**	**—**	**—**	**—**
CRC-specific survival										
No. at risk/events	754/126	165/38	223/63	274/105		712/130	181/32	150/35	106/41	
Model 1 HR (95% CI)^a^	**Ref**	**1.50 (1.03-2.18)**	**1.98 (1.44-2.72)**	**2.92 (2.22-3.86)**	**<0.001**	**Ref**	1.00 (0.67-1.47)	**1.47 (1.02-2.12)**	**2.61 (1.83-3.72)**	**<0.001**
Model 2 HR (95% CI)^b^	**Ref**	1.17 (0.81-1.71)	**1.79 (1.30-2.47)**	**2.70 (2.03-3.59)**	**<0.001**	**—**	**—**	**—**	**—**	**—**

Values shown in bold are statistically significant (*P* value < 0.05).

CI, confidence interval; HR, hazard ratio; Ref, reference; TNM, tumour–node–metastasis.

a Model 1: Adjusted for sex, age, body mass index, alcohol consumption, smoking status, physical exercise, history of cardiovascular disease (heart failure, myocardial infarction, angina pectoris, stroke), history of diabetes, history of hypertension, vitamin D status and season of blood draw.

b Model 2: Adjusted for model 1 + TNM stage.

### Subgroup analyses

Subgroup analyses are presented in Table 3. Associations of higher post-operative CRP levels with lower OS and CSS were rather consistently observed across subgroups defined by sex, age at diagnosis, BMI, vitamin D status and CRC stage (for the DACHS cohort). However, the association of post-operative CRP with OS was much stronger among younger patients (<65 years) than among older patients (*P* value for interaction <0.01 in both cohorts). There was also no statistically significant variation of the prognostic value of post-operative CRP among DACHS cohort patients with stage II or III according to use of adjuvant chemotherapy (all *P* values for interaction >0.05; Supplementary Table S3, available at https://doi.org/10.1016/j.esmoop.2024.102982). Similarly, associations of post-operative CRP with survival outcomes showed no significant variation by tumour location, MS and *BRAF/KRAS* status (all *P* values for interaction >0.05; Supplementary Table S4, available at https://doi.org/10.1016/j.esmoop.2024.102982).

**Table 3:** Multivariable Cox regression results for the association of C-reactive protein levels with survival among subgroups (C-reactive protein <3 mg/l as reference)

Outcome	*n*/events	DACHS cohort			*P*_interaction_	*n*/events	UK Biobank cohort			*P*_interaction_
		3-5 mg/l	5-10 mg/l	≥10 mg/l			3-5 mg/l	5-10 mg/l	≥10 mg/l	
Overall survival										
Male	863/434	1.20 (0.87-1.66)	**1.96 (1.49-2.57)**	**2.14 (1.67-2.75)**	0.47	680/218	1.37 (0.93-2.01)	**2.14 (1.49-3.06)**	**3.13 (2.14-4.58)**	0.44
Female	553/251	0.96 (0.61-1.50)	1.41 (0.95-2.08)	**1.87 (1.32-2.65)**		469/93	1.12 (0.62-2.02)	0.88 (0.44-1.75)	**2.88 (1.52-5.46)**	
<60 years	290/92	0.81 (0.37-1.76)	**1.95 (1.02-3.71)**	**3.15 (1.74-5.73)**	**<0.01**	356/85	1.44 (0.75-2.77)	1.47 (0.76-2.82)	**3.61 (2.01-6.48)**	**<0.01**
60-64 years	246/68	0.76 (0.32-1.81)	**2.80 (1.57-5.00)**	**2.54 (1.41-4.58)**		422/104	1.53 (0.83-2.81)	**2.45 (1.40-4.26)**	**4.27 (2.53-7.20)**	
≥65 years	864/497	1.20 (0.89-1.62)	**1.46 (1.13-1.89)**	**1.63 (1.28-2.06)**		370/122	0.99 (0.62-1.59)	1.32 (0.80-2.17)	1.25 (0.65-2.41)	
Normal weight	536/262	1.08 (0.66-1.74)	**1.79 (1.22-2.61)**	**2.53 (1.83-3.51)**	0.48	292/80	1.38 (0.68-2.80)	**3.17 (1.60-6.28)**	**5.63 (2.89-11.0)**	**<0.01**
Overweight	608/296	1.05 (0.72-1.53)	**1.55 (1.11-2.17)**	**1.38 (1.00-1.90)**		530/125	1.40 (0.87-2.25)	1.60 (0.94-2.72)	**1.91 (1.08-3.36)**	
Obese	272/127	1.34 (0.69-2.60)	**2.10 (1.26-3.51)**	**2.43 (1.45-4.07)**		327/106	0.90 (0.50-1.62)	1.41 (0.83-2.40)	**2.49 (1.46-4.23)**	
Vitamin D deficient	728/284	1.34 (0.69-2.60)	**2.10 (1.26-3.51)**	**2.43 (1.45-4.07)**	0.12	264/93	1.38 (0.77-2.49)	**1.80 (1.02-3.19)**	**3.56 (1.95-6.50)**	0.23
Vitamin D insufficient	408/168	1.45 (0.82-2.56)	**1.85 (1.20-2.84)**	**2.30 (1.51-3.50)**		494/131	1.26 (0.76-2.08)	**1.87 (1.19-2.95)**	**2.65 (1.60-4.39)**	
Vitamin D sufficient	280/117	1.49 (0.85-2.61)	**2.64 (1.51-4.61)**	**2.38 (1.32-4.29)**		391/87	1.19 (0.65-2.19)	0.92 (0.41-2.05)	**2.57 (1.38-4.80)**	
CRC stage I	330/107	1.61 (0.83-3.16)	**1.73 (1.01-2.95)**	**2.14 (1.24-3.68)**	0.30	**—**	**—**	**—**	**—**	**—**
CRC stage II	456/191	1.35 (0.81-2.26)	**2.03 (1.37-3.01)**	**1.92 (1.31-2.81)**		**—**	**—**	**—**	**—**	**—**
CRC stage III	473/243	1.10 (0.73-1.66)	**1.79 (1.20-2.66)**	**1.53 (1.06-2.19)**		**—**	**—**	**—**	**—**	**—**
CRC stage IV	157/138	0.79 (0.40-1.54)	**1.83 (1.06-3.16)**	**2.78 (1.70-4.54)**		**—**	**—**	**—**	**—**	**—**
CRC-specific survival										
Male	863/192	1.07 (0.63-1.80)	**2.36 (1.54-3.60)**	**3.11 (2.12-4.57)**	**0.01**	680/167	1.15 (0.73-1.82)	**1.90 (1.26-2.89)**	**3.21 (2.10-4.91)**	0.59
Female	553/139	1.34 (0.75-2.39)	1.68 (0.98-2.86)	**3.06 (1.98-4.90)**		469/71	0.79 (0.37-1.71)	0.64 (0.28-1.49)	**3.03 (1.48-6.22)**	
<60 years	290/66	0.37 (0.11-1.27)	1.76 (0.77-3.98)	**3.35 (1.75-6.42)**	**<0.01**	356/73	1.27 (0.63-2.57)	1.22 (0.58-2.53)	**2.82 (1.47-5.43)**	**0.02**
60-64 years	246/57	0.83 (0.28-2.49)	**2.86 (1.33-6.18)**	**3.34 (1.45-7.72)**		422/83	1.53 (0.77-3.04)	**2.23 (1.19-4.21)**	**4.47 (2.53-7.91)**	
≥65 years	864/204	1.47 (0.94-2.31)	1.49 (0.98-2.26)	**2.43 (1.69-3.50)**		370/82	0.54 (0.27-1.07)	1.08 (0.58-2.01)	1.25 (0.58-2.68)	
Normal weight	536/123	1.28 (0.63-2.60)	**1.84 (1.00-3.37)**	**3.66 (2.22-6.01)**	0.48	292/65	1.21 (0.53-2.76)	**2.71 (1.24-5.91)**	**5.72 (2.82-11.6)**	**0.02**
Overweight	608/130	1.23 (0.70-2.14)	**2.16 (1.35-3.45)**	**2.17 (1.38-3.41)**		530/96	1.02 (0.56-1.86)	1.43 (0.77-2.64)	**2.22 (1.20-4.11)**	
Obese	272/69	0.78 (0.29-2.08)	1.31 (0.60-2.85)	**2.12 (1.04-4.33)**		327/77	0.77 (0.39-1.55)	1.16 (0.62-2.18)	**1.99 (1.06-3.74)**	
Vitamin D deficient	728/199	0.96 (0.56-1.65)	**1.59 (1.03-2.47)**	**2.66 (1.84-3.85)**	0.54	264/65	1.12 (0.54-2.31)	1.46 (0.73-2.93)	**2.58 (1.24-5.36)**	0.81
Vitamin D insufficient	408/75	**2.25 (1.00-5.06)**	**2.59 (1.30-5.14)**	**4.42 (2.34-8.34)**		494/110	1.09 (0.61-1.95)	**1.73 (1.04-2.86)**	**2.81 (1.65-4.77)**	
Vitamin D sufficient	280/58	0.98 (0.41-2.35)	**3.64 (1.70-7.78)**	**3.98 (1.69-9.35)**		391/63	0.82 (0.36-1.87)	0.68 (0.24-1.91)	**2.74 (1.36-5.49)**	
CRC stage I	330/21	1.05 (0.11-9.88)	**3.87 (1.04-14.4)**	**10.0 (3.05-33.4)**	0.90	**—**	**—**	**—**	**—**	**—**
CRC stage II	456/57	1.77 (0.72-4.34)	2.10 (0.98-4.52)	2.03 (0.99-4.17)		**—**	**—**	**—**	**—**	**—**
CRC stage III	473/124	1.41 (0.82-2.50)	**2.54 (1.49-4.35)**	**2.44 (1.49-4.00)**		**—**	**—**	**—**	**—**	**—**
CRC stage IV	157/126	0.77 (0.38-1.53)	1.48 (0.83-2.64)	**2.51 (1.51-4.15)**		**—**	**—**	**—**	**—**	**—**

Cox regression analyses for both cohorts were adjusted for sex, age, body mass index, alcohol consumption, smoking status, physical exercise, history of cardiovascular disease (heart failure, myocardial infarction, angina pectoris, stroke), history of diabetes, history of hypertension, vitamin D status and season of blood draw. DACHS cohort analyses were additionally adjusted for TNM stage. Cox regression results are presented as HRs and 95% CIs. Values shown in bold are statistically significant (*P* value < 0.05).

CI, confidence interval; HR, hazard ratio; TNM, tumour–node–metastasis.

### Sensitivity analyses

Sensitivity analyses in which follow-up time was restricted to a maximum of 5 years after blood draw showed even stronger dose–response relationships between elevated post-operative CRP concentrations and both survival outcomes (Supplementary Table S5, available at https://doi.org/10.1016/j.esmoop.2024.102982).

## Discussion

Our results showed strong and independent dose–response relationships between elevated serum levels of CRP assessed a month or more after surgery and worse OS and CSS. The association of CRP with OS was particularly strong among male patients and also younger patients <65 years of age.

CRP is a highly sensitive marker of systemic inflammation and it is primarily produced by liver hepatocytes in response to elevated inflammatory cytokines such as interleukin-6.^24^ The exact mechanism by which CRP is related to the prognosis of patients with CRC remains obscure, but mechanistic studies have suggested that elevated CRP correlates with increased expression of oncogenes resulting in DNA damage.^25^ Consequently, elevated circulating CRP has been widely reported as a marker of poor prognosis,^11^^,^^26^ infectious complications^27^ and compromised treatment response^22^ in patients with various disease conditions including cancer. Indeed, we observed positive associations between post-operative serum CRP levels and disease relapse, which might explain the shorter OS and CSS in our study, particularly for patients with CRP >5 mg/l.

While the clinical relevance of modestly increased CRP detectable by high-sensitivity CRP measurements (e.g. CRP between 3 and 10 mg/l) remains elusive for cancer patients, levels <3 mg/l are generally known to be reflective of good health and disease control.^22^ Very few and mostly smaller studies have previously assessed the associations of post-operative CRP levels and prognosis among CRC patients. A most recent systematic review and meta-analysis of these studies likewise found elevated levels of CRP in blood samples taken 1 or more months after surgery to be associated with poorer OS and CSS.^28^ However, due to the low number and strong heterogeneity in the design of the studies, and their limited sample size, meta-analyses did not allow comprehensive analyses of dose–response patterns which are clearly addressed in our study.

In the subgroup analyses, our results showed stronger associations of post-operative CRP with OS for younger compared to older patients. More aggressive CRC has been reported in younger populations partly due to late-stage diagnosis linked to a lack of early detection guidelines for younger populations.^29^ The recent increase in the global burden of early-onset CRC underscores the need for targeted early detection and intervention programmes in younger populations.^30^, ^31^, ^32^ In addition, the association between elevated CRP and survival in older patients could be confounded by several factors including age-related comorbidities.^33^^,^^34^ Male compared to female sex was generally a risk factor for worse prognosis among CRC patients with elevated post-operative CRP levels. Male patients may have poor health outcomes compared to female patients partly attributed to more frequent high-risk behaviour, for example, excessive smoking, high alcohol consumption and poor health care seeking behaviour.^35^ The ‘obesity paradox’ is confirmed by our findings in which we showed post-diagnostic obesity as a predictor of better survival outcomes among CRC patients.^36^^,^^37^

We did not find any significant interaction between post-operative CRP and vitamin D status in the prediction of survival outcomes although preclinical evidence consistently reports the role of vitamin D in immune-inflammatory modulation.^38^^,^^39^ The prognostic role of post-operative serum vitamin D levels for both OS and CSS has been shown to be independent of CRP levels.^40^ Although previous studies have reported chemotherapy-induced inflammation in cancer patients,^41^^,^^42^ we did not observe any significant interaction between chemotherapy use and CRP levels, probably due to low statistical power. Moreover, CRP showed prognostic value for survival irrespective of tumour location and *BRAF/KRAS* status.

### Limitations and future research

Despite the large overall sample size of the patient cohorts, case numbers were low for some of the subgroup analyses. Furthermore, there was substantial heterogeneity in the timing of blood draw for CRP measurements after surgery (mostly within a few weeks to months after surgery). However, the consistency of our results despite this variation suggests that CRP assessed a month or more after surgery may be a relevant prognostic marker regardless of the exact timing of assessment. While there is emerging evidence on the prognostic value of circulating tumour DNA (ctDNA) among resected CRC patients,^43^, ^44^, ^45^ ctDNA was not assessed in our cohorts. Nonetheless, we think that CRP, which showed similar or even stronger prognostic value compared to reported prognostic value of ctDNA,^46^ could be a rather routine and cheaper prognostic biomarker to apply in clinical settings as compared to the more expensive ctDNA, especially for low-resource settings. Variables for post-operative complications were also not available in our study cohorts, but a previous study demonstrated that CRP assessed within a month after surgery was a prognostic factor for CSS, independent of post-operative complications.^47^ Future studies should establish the prognostic potential of post-operative CRP in combination with these emerging or established prognostic factors and provide risk-stratifying criteria for surgical CRC patients.

### Conclusions

Serum CRP determined a month or more after surgery may be useful as a prognostic biomarker and for surveillance of the course of disease of CRC patients, particularly younger patients <65 years of age. Future studies should validate our findings and evaluate the potential use of post-operative serum CRP in surveillance and treatment decisions in the long-term care of CRC patients.
