# Supplementary material for: Post-operative C-reactive protein as a strong independent predictor of long-term colorectal cancer outcomes: consistent findings from two large patient cohorts
Source: ESMO Open. 2024 Apr 12;9(4):102982. doi: 10.1016/j.esmoop.2024.102982 (PMC11033061; doi:10.1016/j.esmoop.2024.102982)
Supplement: Supplementary data [file mmc1.docx]

**Table of Contents**

[**Supp. Fig. 1** Distribution of post-operative C-reactive protein by time of blood collection after surgery for the DACHS cohort (panel A) and the UK Biobank cohort (panel B). 2](#_Toc158024634)

[**Supp. Fig. 2** Overall (panel A and B) and CRC-specific (panel C and D) survival by post-operative C-reactive protein categories. 3](#_Toc158024635)

[**Supp. Table 1** Cox regression associations of post-operative C-reactive protein with relapse-free survival in the DACHS cohort 4](#_Toc158024636)

[**Supp. Table 2** Cox regression associations of post-operative C-reactive protein with survival in the UK Biobank cohort after additional adjustment for blood-cell count based biomarkers. 5](#_Toc158024637)

[**Supp. Table 3** Cox regression associations of C-reactive protein levels with survival among TNM stage II and III patients in the DACHS cohort 6](#_Toc158024638)

[**Supp. Table 4** Cox regression associations of C-reactive protein levels with survival among selected DACHS cohort subgroups. 7](#_Toc158024639)

[**Supp. Table 5** Association of C-reactive with 5-year survival outcomes for the DACHS and UK Biobank cohorts 8](#_Toc158024640)

# **Supp. Fig. 1** Distribution of post-operative C-reactive protein by time of blood collection after surgery for the DACHS cohort (panel A) and the UK Biobank cohort (panel B).

| **A.** | **B.** |
| --- | --- |
| 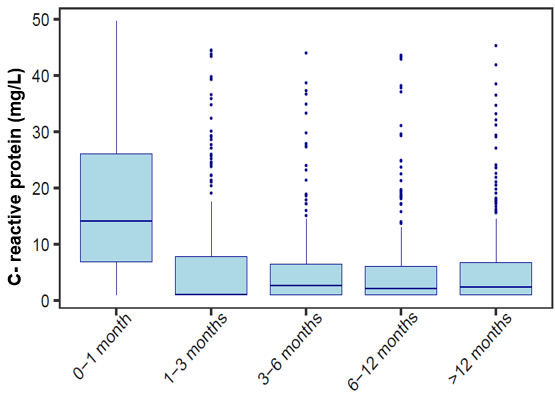 | 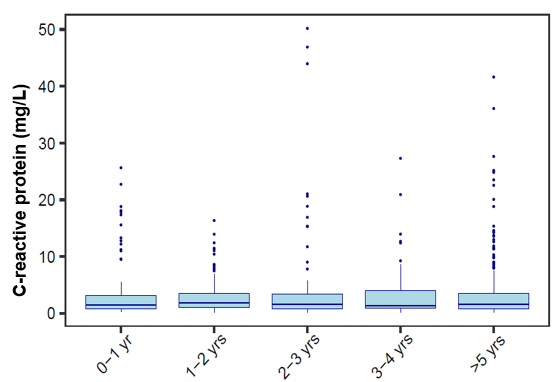 |

**Notes**: In the DACHS cohort final main analyses, patients with blood samples collected within the first month post-surgery were excluded.

# **Supp. Fig. 2** Overall (panel A and B) and CRC-specific (panel C and D) survival by post-operative C-reactive protein categories.

| 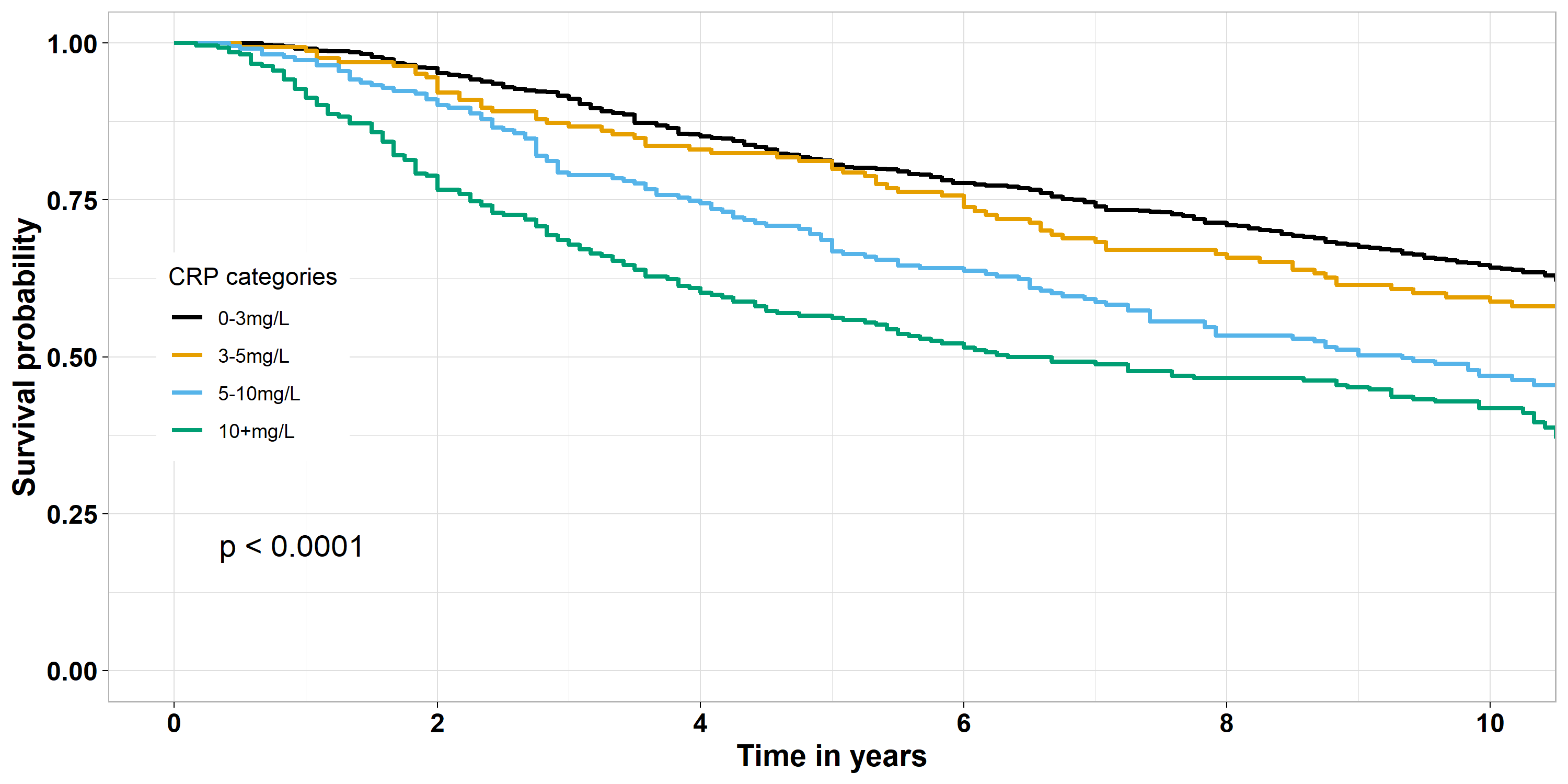 | 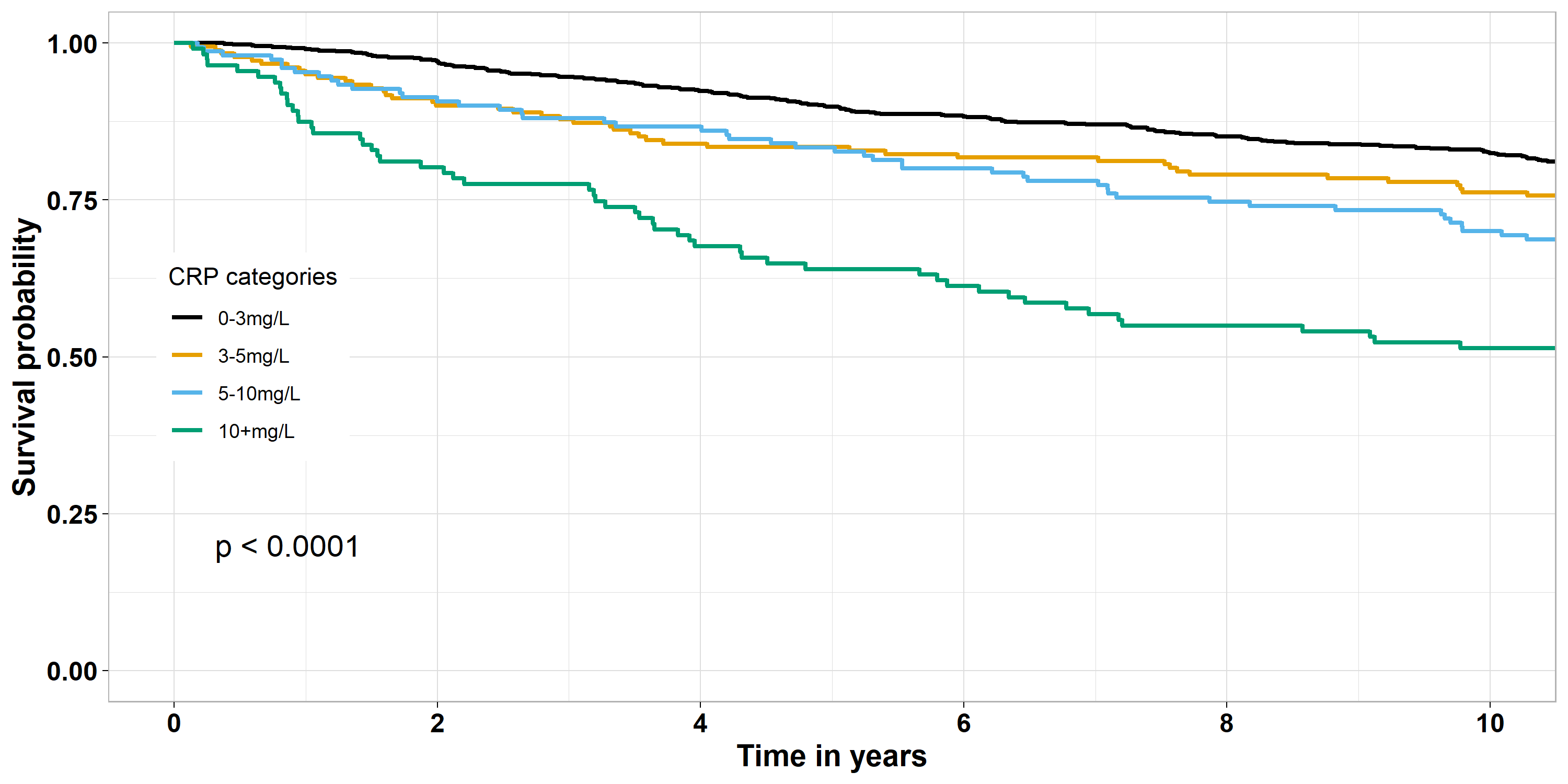 |
| --- | --- |
| 1. DACHS cohort | 1. UK Biobank cohort |
| 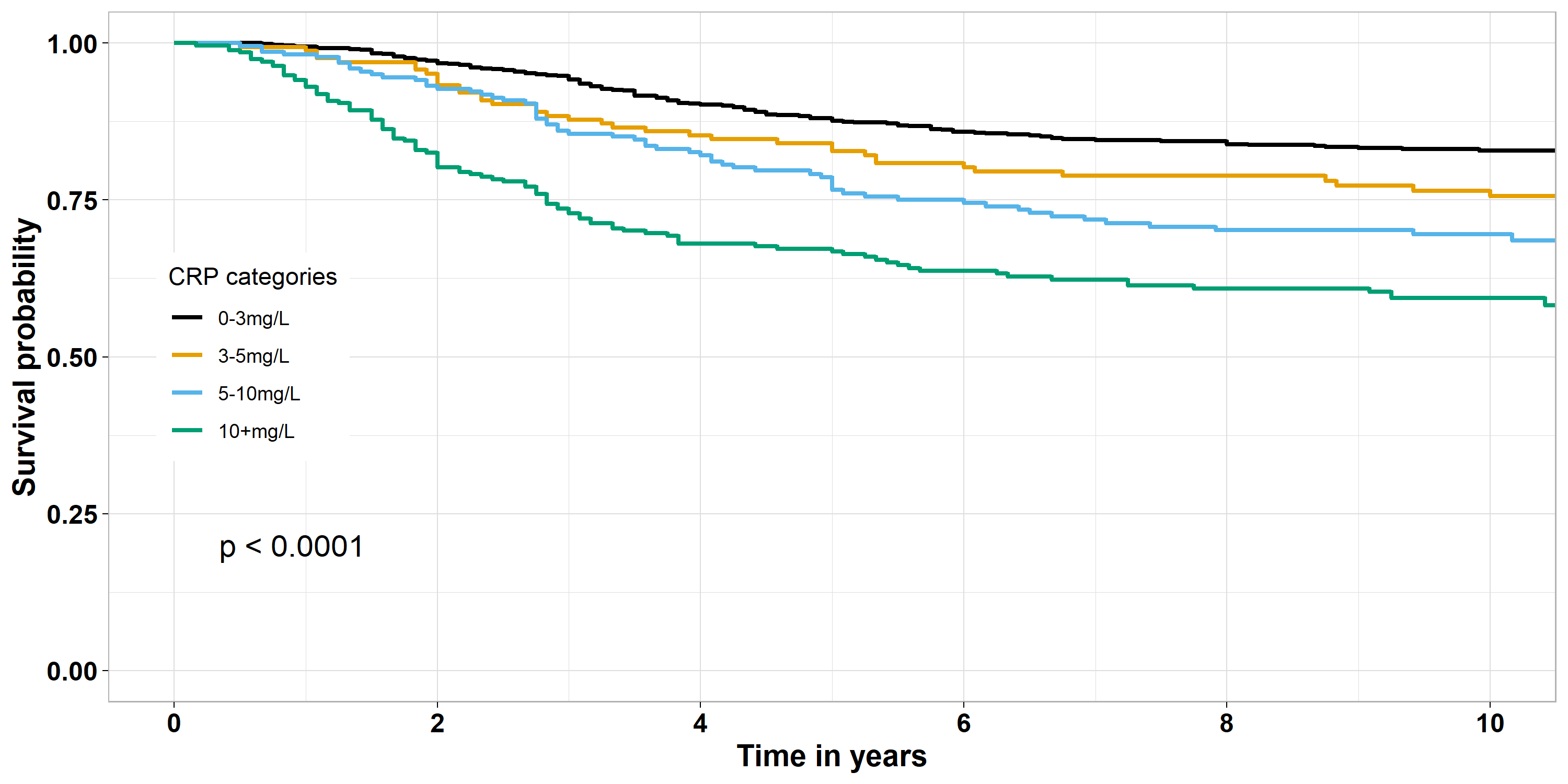 | 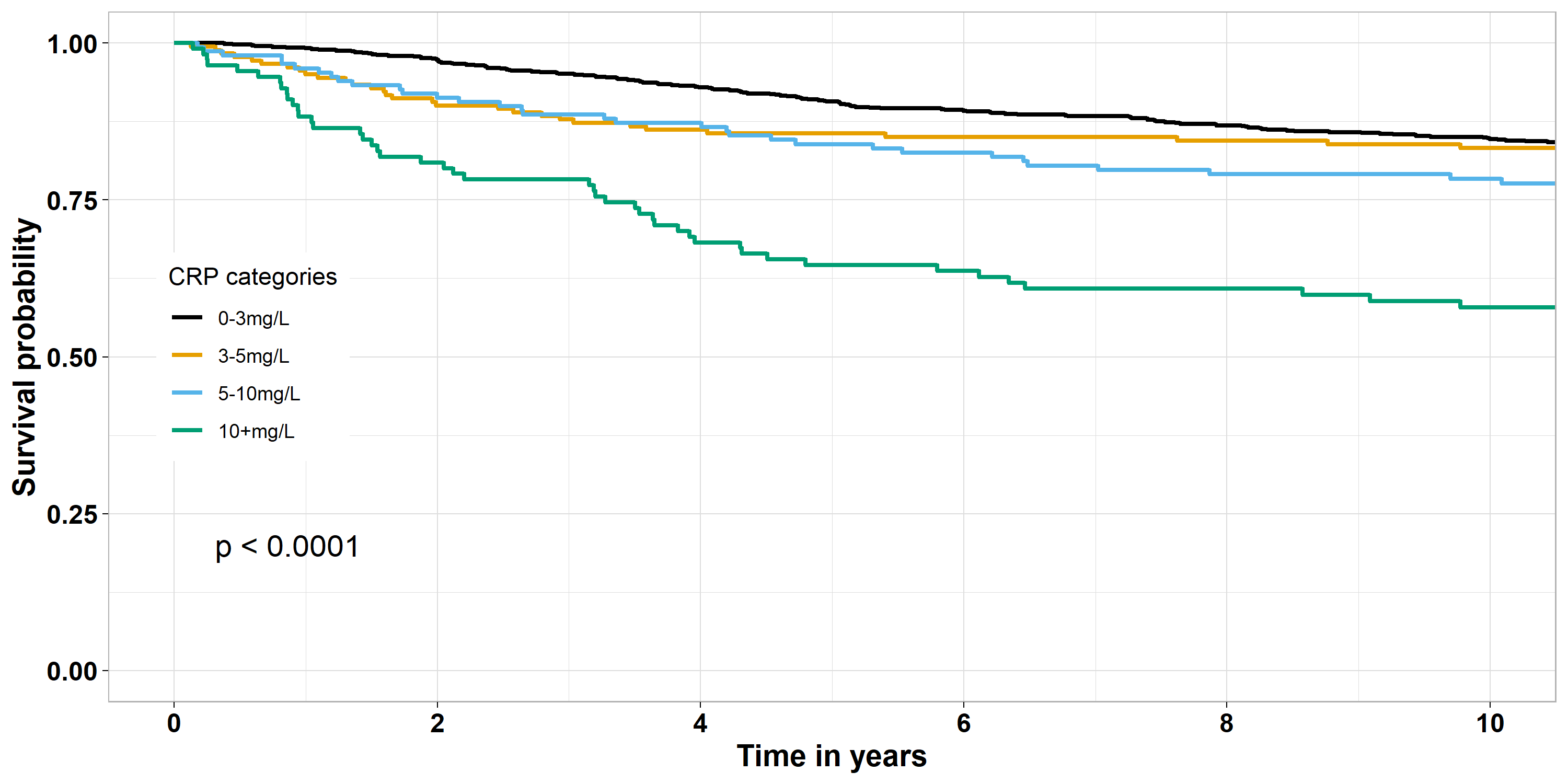 |
| 1. DACHS cohort | 1. UK Biobank cohort |

# **Supp. Table 1** Cox regression associations of post-operative C-reactive protein with relapse-free survival in the DACHS cohort

| **OUTCOME** | **DACHS cohort** | | | | **P-trend** |
| --- | --- | --- | --- | --- | --- |
|  | **C-reactive protein serum level** | | | |  |
|  | **<3 mg/L** | **3 – 5 mg/L** | **5 – 10 mg/L** | **≥10 mg/L** |  |
| **Relapse-free survival** |  |  |  |  |  |
| No. at risk/events | 754/170 | 165/49 | 223/80 | 274/108 |  |
| Model 1 HR (95% CI)**†** | **Ref** | **1.41 (1.02 – 1.96)** | **1.81 (1.37 – 2.39)** | **2.13 (1.65 – 2.75)** | **<0.001** |
| Model 2 HR (95% CI)**ǂ** | **Ref** | 1.13 (0.82 – 1.58) | **1.66 (1.26 – 2.20)** | **1.73 (1.33 – 2.25)** | **<0.001** |

**Abbreviations:** CI, confidence interval; HR, hazard ratio; Ref, reference.

**†Model 1:** Adjusted for ~ sex, age, body mass index, alcohol consumption, smoking status, physical exercise, history of cardiovascular disease (heart failure, myocardial infarction, angina pectoris, stroke), history of diabetes, history of hypertension, vitamin D status, and season of blood draw.

**ǂModel 2:** Adjusted for ~ Model 1 + cancer stage

# **Supp. Table 2** Cox regression associations of post-operative C-reactive protein with survival in the UK Biobank cohort after additional adjustment for blood-cell count based biomarkers.

| **OUTCOME** | **DACHS cohort** | | | | **P_trend_** |
| --- | --- | --- | --- | --- | --- |
|  | **C-reactive protein serum level** | | | |  |
|  | **<3 mg/L** | **3 – 5 mg/L** | **5 – 10 mg/L** | **≥10 mg/L** |  |
| **Overall survival** |  |  |  |  |  |
| No. at risk/events | 712/159 | 181/50 | 150/51 | 106/51 |  |
| Model 1 HR (95% CI)**†** | **Ref** | 1.26 (0.92 – 1.74) | **1.69 (1.23 – 2.31)** | **2.70 (1.96 – 3.71)** | **<0.001** |
| Model 2 HR (95% CI)**ǂ** | **Ref** | 1.21 (0.88 – 1.67) | **1.61 (1.17 – 2.21)** | **2.54 (1.84 – 3.52)** | **<0.001** |
|  |  |  |  |  |  |
| **Cancer-specific survival** |  |  |  |  |  |
| No. at risk/events | 712/130 | 181/32 | 150/35 | 106/41 |  |
| Model 1 HR (95% CI)**†** | **Ref** | 1.00 (0.67 – 1.47) | **1.47 (1.02 – 2.12)** | **2.61 (1.83 – 3.72)** | **<0.001** |
| Model 2 HR (95% CI)**ǂ** | **Ref** | 0.98 (0.66 – 1.46) | **1.43 (1.00 – 2.08)** | **2.51 (1.75 – 3.61)** | **<0.001** |
|  |  |  |  |  |  |

**Abbreviations:** CI, confidence interval; HR, hazard ratio; Ref, reference.

**†Model 1:** Adjusted for ~ sex, age, body mass index, alcohol consumption, smoking status, physical exercise, history of cardiovascular disease (heart failure, myocardial infarction, angina pectoris, stroke), history of diabetes, history of hypertension, vitamin D status, and season of blood draw.

**ǂModel 2:** Adjusted for ~ Model 1 + Lymphocyte/Monocyte Ratio + Platelet/Lymphocyte Ratio + Neutrophil/Lymphocyte Ratio

# **Supp. Table 3** Cox regression associations of C-reactive protein levels with survival among TNM stage II and III patients in the DACHS cohort

| **Outcome** | **N/events** | **TNM Stage II** | | | **P_interaction_** |
| --- | --- | --- | --- | --- | --- |
|  |  | **CRP: 3 - 5mg/L** | **CRP: 5 - 10mg/L** | **CRP: ≥10mg/L** |  |
| **Overall Survival** |  |  |  |  |  |
| No chemotherapy | 347/147 | 1.16 (0.61 – 2.20) | **2.00 (1.28 – 3.11)** | **1.66 (1.06 – 2.61)** | 0.52 |
| Adj. chemotherapy | 109/42 | 1.72 (0.58 – 5.11) | 1.88 (0.64 – 5.53) | **4.72 (1.79 – 12.46)** |  |
| **CRC-specific survival** |  |  |  |  |  |
| No chemotherapy | 347/39 | 1.74 (0.59 – 5.17) | 1.95 (0.78 – 4.88) | 1.32 (0.51 – 2.74) | 0.13 |
| Adj. chemotherapy | 109/18 | 1.45 (0.21 – 9.92) | 2.82 (0.44 – 18.24) | **10.10 (2.16 – 46.78)** |  |
| **Relapse-free survival** |  |  |  |  |  |
| No chemotherapy | 347/58 | 1.03 (0.41 – 2.60) | 1.28 (0.63 – 2.61) | 0.71 (0.30 – 1.66) | 0.34 |
| Adj. chemotherapy | 109/23 | 0.37 (0.06 – 2.19) | 1.30 (0.29 – 5.71) | 2.51 (0.71 – 8.93) |  |
| **TNM Stage III** | | | | | |
| **Overall Survival** |  |  |  |  |  |
| No chemotherapy | 68/51 | 0.81 (0.20 – 3.30) | 1.36 (0.47 – 3.99) | 1.60 (0.61 – 4.20) | 0.54 |
| Adj. chemotherapy | 405/190 | 1.22 (0.78 – 1.89) | **1.85 (1.16 – 2.93)** | **1.50 (1.00 – 2.28)** |  |
| **CRC-specific survival** |  |  |  |  |  |
| No chemotherapy | 68/21 | 6.12 (0.99 – 38.19) | 2.45 (0.39 – 15.35) | 4.54 (0.71 – 29.24) | 0.70 |
| Adj. chemotherapy | 405/101 | 1.38 (0.73 – 2.58) | **2.75 (1.53 – 4.94)** | **2.51 (1.46 – 4.34)** |  |
| **Relapse-free survival** |  |  |  |  |  |
| No chemotherapy | 68/27 | 2.15 (0.39 – 11.74) | 1.29 (0.28 – 5.85) | 2.31 (0.54 – 9.83) | 0.63 |
| Adj. chemotherapy | 405/126 | 1.66 (0.97 – 2.84) | **2.81 (1.67 – 4.73)** | **2.11 (1.26 – 3.53)** |  |
|  |  |  |  |  |  |

**Abbreviations**: Adj., adjuvant; CRC, colorectal cancer; CRP, c-reactive protein; TNM, tumor-node-metastasis
**Notes**: Hazard Ratios with 95% Confidence Intervals were computed with CRP <3mg/L as reference category; Cox regressions were adjusted for ~ sex, age at diagnosis, body mass index, alcohol consumption, smoking status, physical exercise, history of cardiovascular disease (heart failure, myocardial infarction, angina pectoris, stroke), history of diabetes, history of hypertension, vitamin D status, and season of blood draw.

# **Supp. Table 4** Cox regression associations of C-reactive protein levels with survival among selected DACHS cohort subgroups.

| **Outcome** | **N/events** | **DACHS cohort** | | | **P_interaction_** |
| --- | --- | --- | --- | --- | --- |
|  |  | **CRP: 3 - 5mg/L** | **CRP: 5 - 10mg/L** | **CRP: ≥10mg/L** |  |
| **Overall Survival** |  |  |  |  |  |
| MSS | 873/453 | 1.08 (0.79 – 1.46) | **1.65 (1.26 – 2.15)** | **2.09 (1.64 – 2.68)** | 0.07 |
| MSI | 106/40 | **0.11 (0.01 – 0.96)** | 1.42 (0.50 – 4.03) | 0.73 (0.29 – 1.83) |  |
|  |  |  |  |  |  |
| BRAF wild type | 944/470 | 1.00 (0.74 – 1.36) | **1.55 (1.20 – 2.01)** | **2.00 (1.57 – 2.53)** | 0.87 |
| BRAF mutant | 84/44 | 3.27 (0.89 – 11.9) | **3.43 (1.12 – 10.5)** | **3.42 (1.10 – 10.6)** |  |
|  |  |  |  |  |  |
| KRAS wild type | 676/335 | 1.15 (0.79 – 1.68) | **1.54 (1.12 – 2.11)** | **2.11 (1.60 – 2.78)** | 0.84 |
| KRAS mutant | 354/179 | 1.08 (0.65 – 1.79) | **1.71 (1.10 – 2.65)** | **1.69 (1.10 – 2.59)** |  |
|  |  |  |  |  |  |
| No chemotherapy | 749/324 | 1.25 (0.82 – 1.90) | **1.72 (1.27 – 2.32)** | **1.58 (1.16 – 2.14)** | 0.09 |
| Adj. chemotherapy | 667/362 | 1.04 (0.74 – 1.46) | **1.54 (1.11 – 2.14)** | **2.31 (1.75 – 3.04)** |  |
|  |  |  |  |  |  |
| Right-sided CRC | 462/232 | 0.88 (0.55 – 1.43) | **1.92 (1.30 – 2.83)** | **1.82 (1.29 – 2.59)** | 0.44 |
| Left-sided CRC | 886/420 | 1.19 (0.86 – 1.65) | **1.61 (1.22 – 2.13)** | **2.10 (1.62 – 2.72)** |  |
| **CRC-specific survival** |  |  |  |  |  |
| MSS | 873/239 | 1.22 (0.80 – 1.86) | **1.84 (1.25 – 2.69)** | **2.73 (1.95 – 3.84)** | n. a |
| MSI | 106/8 | n. a | n. a | n. a |  |
|  |  |  |  |  |  |
| BRAF wild type | 944/235 | 1.08 (0.70 – 1.68) | **1.63 (1.12 – 2.39)** | **2.63 (1.88 – 3.68)** | 0.30 |
| BRAF mutant | 84/18 | 22.7 (0.91 – 570.1) | 13.7 (0.80 – 233.9) | **80.1 (4.25 – 1508.4)** |  |
|  |  |  |  |  |  |
| KRAS wild type | 676/151 | **1.83 (1.08 – 3.09)** | **1.96 (1.23 – 3.14)** | **3.13 (2.09 – 4.68)** | 0.63 |
| KRAS mutant | 354/94 | 0.80 (0.39 – 1.66) | 1.56 (0.85 – 2.89) | **1.88 (1.04 – 3.40)** |  |
|  |  |  |  |  |  |
| No chemotherapy | 749/96 | 1.84 (0.87 – 3.90) | 1.78 (0.97 – 3.26) | **2.35 (1.33 – 4.17)** | 0.66 |
| Adj. chemotherapy | 667/235 | 1.03 (0.66 – 1.60) | **1.80 (1.21 – 2.68)** | **2.87 (2.04 – 4.04)** |  |
|  |  |  |  |  |  |
| Right-sided CRC | 462/98 | 1.14 (0.54 – 2.45) | **2.50 (1.34 – 4.66)** | **2.85 (1.66 – 4.87)** | 0.86 |
| Left-sided CRC | 886/217 | 1.09 (0.69 – 1.73) | **1.76 (1.17 – 2.64)** | **2.76 (1.92 – 3.95)** |  |
| **Relapse-free survival** |  |  |  |  |  |
| MSS | 873/285 | 1.23 (0.84 – 1.79) | **1.61 (1.14 – 2.27)** | **1.87 (1.37 – 2.55)** | 0.70 |
| MSI | 106/11 | n. a | 1.58 (0.32 – 7.89) | 1.97 (0.54 – 7.14) |  |
|  |  |  |  |  |  |
| BRAF wild type | 944/289 | 1.14 (0.78 – 1.67) | **1.43 (1.02 – 2.00)** | **1.78 (1.31 – 2.42)** | 0.38 |
| BRAF mutant | 84/21 | 15.3 (0.72 – 325.8) | **25.6 (1.62 – 403.7)** | **70.1 (3.55 – 1386.7)** |  |
|  |  |  |  |  |  |
| KRAS wild type | 676/196 | 1.31 (0.81 – 2.11) | **1.65 (1.09 – 2.49)** | **1.99 (1.38 – 2.87)** | 0.84 |
| KRAS mutant | 354/110 | 1.31 (0.70 – 2.44) | 1.29 (0.73 – 2.27) | 1.58 (0.91 – 2.76) |  |
|  |  |  |  |  |  |
| No chemotherapy | 749/132 | 1.43 (0.77 – 2.65) | 1.51 (0.93 – 2.47) | 1.48 (0.91 – 2.42) | 0.63 |
| Adj. chemotherapy | 667/275 | 1.05 (0.70 – 1.56) | **1.70 (1.19 – 2.43)** | **1.80 (1.31 – 2.49)** |  |
|  |  |  |  |  |  |
| Right-sided CRC | 462/112 | 0.91 (0.45 – 1.85) | **1.86 (1.06 – 3.24)** | **1.93 (1.19 – 3.15)** | 0.24 |
| Left-sided CRC | 886/277 | 1.18 (0.80 – 1.75) | **1.72 (1.22 – 2.43)** | **1.73 (1.24 – 2.41)** |  |

**Abbreviations**: CRC, colorectal cancer; CRP, c-reactive protein; MSS, microsatellite stable; MSI, microsatellite instable; n. a, not applicable (no events were recorded); TNM, tumor-node-metastasis.

**Notes**: Hazard Ratios with 95% Confidence Intervals were computed with CRP <3mg/L as reference category; Cox regressions were adjusted for ~ sex, age at diagnosis, stage at diagnosis, body mass index, alcohol consumption, smoking status, physical exercise, history of cardiovascular disease (heart failure, myocardial infarction, angina pectoris, stroke), history of diabetes, history of hypertension, vitamin D status, and season of blood draw.

# **Supp. Table 5** Association of C-reactive with 5-year survival outcomes for the DACHS and UK Biobank cohorts

| **OUTCOME** | **DACHS cohort** | | | | **P-trend** | **UK Biobank cohort** | | | | **P-trend** |
| --- | --- | --- | --- | --- | --- | --- | --- | --- | --- | --- |
|  | **C-reactive protein serum level** | | | |  | **C-reactive protein serum level** | | | |  |
|  | **<3 mg/L** | **3 – 5 mg/L** | **5 – 10 mg/L** | **≥10 mg/L** |  | **<3 mg/L** | **3 – 5 mg/L** | **5 – 10 mg/L** | **≥10 mg/L** |  |
| **Overall survival** |  |  |  |  |  |  |  |  |  |  |
| Number at risk/events | 754/112 | 165/24 | 223/59 | 273/105 |  | 712/75 | 181/30 | 150/25 | 106/36 |  |
| Adj. HR (95% CI)**†** | **Ref** | 0.92 (0.59 – 1.44) | **1.88 (1.35 – 2.61)** | **2.97 (2.22 – 3.97)** | **<0.001** | **Ref** | **1.68 (1.09 – 2.58)** | **1.67 (1.05 – 2.65)** | **3.62 (2.41 – 5.43)** | **<0.001** |
|  |  |  |  |  |  |  |  |  |  |  |
| **CRC-specific survival** |  |  |  |  |  |  |  |  |  |  |
| No. at risk/events | 754/75 | 165/22 | 223/40 | 273/85 |  | 712/72 | 181/26 | 150/24 | 106/35 |  |
| Adj. HR (95% CI)**†** | **Ref** | 1.09 (0.67 – 1.77) | **1.86 (1.26 – 2.77)** | **3.36 (2.39 – 4.73)** | **<0.001** | **Ref** | 1.53 (0.97 – 2.40) | **1.66 (1.04 – 2.66)** | **3.64 (2.41 – 5.50)** | **<0.001** |

**Abbreviations:** CI, confidence interval; CRC, colorectal cancer; HR, hazard ratio; Ref, reference.

**†** All cohorts were adjusted for ~ sex, age at diagnosis, body mass index, alcohol consumption, smoking status, physical exercise, history of cardiovascular disease (heart failure, myocardial infarction, angina pectoris, stroke), history of diabetes, history of hypertension, vitamin D status, and season of blood draw (DACHS cohort was additionally adjusted for cancer stage).
